# Maintenance of androgen deprivation therapy or testosterone supplementation in the management of castration-resistant prostate cancer: that is the question

**DOI:** 10.1007/s12020-022-03166-w

**Published:** 2022-08-20

**Authors:** Irene Caramella, Alberto Dalla Volta, Marco Bergamini, Deborah Cosentini, Francesca Valcamonico, Alfredo Berruti

**Affiliations:** Medical Oncology Unit, Department of Medical and Surgical Specialties, Radiological Sciences, and Public Health, University of Brescia. ASST Spedali Civili, Brescia, Italy

**Keywords:** Castration resistant prostate cancer, Androgen deprivation therapy, Testosterone, Bipolar androgen therapy

## Abstract

**Purpose:**

Whether or not androgen receptor (AR) axis could still be targetable in castration resistant prostate cancer (CRPC) patients with disease progression to next generation hormonal agents (NGHAs) is a controversial issue.

**Results:**

Serum testosterone in CRPC patients has a positive prognostic role and increasing testosterone levels after androgen deprivation therapy (ADT) withdrawal or testosterone supplementation, as part of a bipolar androgen therapy (BAT) strategy, has been shown to potentially restore sensitivity to previous lines of NGHAs.

**Conclusion:**

These data suggest that maintenance of ADT in CRPC patients receiving further lines of treatment, as recommended by current international guidelines, could be questionable. Conversely, testosterone supplementation aimed to re-sensitize CRPC to further hormonal manipulation is a strategy worth to be explored in future clinical trials.

Prostate cancer is an androgen-dependent disease and androgen deprivation therapy (ADT) is the mainstay of treatment for relapsed or metastatic patients. The biology of castration resistant prostate cancer (CRPC) still depends on androgen receptor (AR) signaling through AR gene amplification, overexpression, and production of ligand-independent variants [1]. This implies that patients with CRPC frequently obtain a consistent benefit from the administration of next generation hormonal agents (NGHAs) such as enzalutamide, abiraterone, apalutamide, and darolutamide. Upon progression to these drugs, however, the therapeutic relevance of AR targeting in further disease management seems to be elusive, since retrospective [2] as well as prospective [3] clinical data show that NGHAs in this setting are poorly effective, with an estimated overall response rate of 12–13%.

Recently, on the basis of the results of several prospective randomized clinical trials, that have demonstrated a remarkable efficacy of NGHAs in HSPC patients, the current use of these drugs has moved from CRPC to HSPC setting. So CRPC phenotype has changed and NGHAs will no longer be effective in this context.

Whereas newer treatment strategies are focused on targets beyond the AR (i.e., PARP-inhibitors, radioligands, and immunotherapy), international guidelines still recommend the maintenance of castrate levels of testosterone in pretreated CRPC patients [4]. This recommendation, however, is based on questionable evidence, derived from a single retrospective study showing a modest advantage in overall survival for patients maintaining ADT in association with an outdated chemotherapy regimen [5]. Noteworthy, this survival benefit was not confirmed in 4 subsequent retrospective studies [6–9].

Based on these considerations, is there still a role for castration in CRPC patients receiving AR independent treatments upon progression to NGHAs?

A meta-analysis by Claps et al. [10] showed that the correlation between serum testosterone and prostate cancer prognosis varies in different clinical settings across the natural history of the disease.

The authors observed an inverse relationship between serum testosterone concentrations and patient prognosis, either in terms of progression-free survival (PFS) or OS, in metastatic HSPC patients after few months of ADT. Conversely, in CRPC patients, higher testosterone levels were associated with longer PFS and OS, regardless the type of treatment received (NGHAs or docetaxel).

The observed positive prognostic effect of testosterone levels in CRPC patients may question the appropriateness of maintaining ADT in this phase of the disease, mostly when associated with non-hormonal treatments such as chemotherapy.

This issue was addressed in the PON-PC study, a recently published clinical trial where CRPC patients were randomized to receive docetaxel with or without ADT maintenance [11]. The results showed no difference in efficacy outcomes (OS, radiological and biochemical PFS) between the two arms.

Unfortunately, the generalization of the study results was hampered by 2 major limitations: (1) the study was early interrupted when 1/3 of planned patients were enrolled, (2) only 7% of patients randomized to ADT withdrawal achieved a serum testosterone level >0.5 ng/ml in the off-therapy phase, in contrast with the reported time to testosterone normalization of about 3 months in HSPC patients in the off phase of intermittent ADT schedules [12]. Testicular atrophy, due to the long-term ADT exposure in the majority of the PON-PC patients, could be a plausible explanation for this phenomenon [13–15].

These limitations notwithstanding, PON-PC study found that patients randomized to discontinuation of ADT, achieving testosterone levels above the castration range, did not have a worse prognosis than their counterpart. Conversely, a non-significant survival increase of 4 months was observed in this subgroup, in accordance with the results of the meta-analysis by Claps et al. [10].

The results of the PON-PC trial clearly demonstrate that most CRPC patients do not undergo complete testosterone restoration upon ADT withdrawal alone, suggesting that a testosterone replacement therapy is required [16].

Indeed, efficacy and safety of testosterone supplementation in CRPC setting were investigated in studies exploring the so-called bipolar androgen therapy (BAT).

BAT is a therapeutic strategy based on periodic administration of injective testosterone in combination with ADT [17]. The resulting alternative supraphysiological and near-castrate hormonal concentrations exert an antiproliferative activity through impaired regulation of AR expression in response to hormonal fluctuations and subsequent disruption of DNA relicensing required for cell division [18]. BAT, as a single antineoplastic therapy, demonstrated a clinically significant activity both in terms of PSA response and disease control in four single-arm phase I/II studies involving pre-treated CRPC patients [19].

The first randomized clinical trial with BAT (TRANSFORMER), recently published by Denmeade et al., compared BAT with enzalutamide in CRPC patients progressing on abiraterone and showed similar efficacy outcomes for the two apparently opposed therapeutic strategies [20]. Of note, health-related quality of life (HRQoL) and patients reported outcomes (PROs) significantly favored BAT compared to enzalutamide, hinting at a potential clinical benefit of testosterone restoration in terms of fatigue, sexual dysfunction and eventually other hypogonadism-related metabolic toxicities [21–23].

These data are in line with previously cited phase I/II studies and clearly show that testosterone can be safely administered to CRPC patients, with the potential to achieve disease response and a consistent improvement in HRQoL.

Back to PON-PC trial, another relevant phenomenon was observed in patients experiencing hormonal restoration: 4 study subjects, whose testosterone serum concentrations reached normal levels upon luteinizing hormone-releasing hormone agonist (LHRHa) withdrawal, were described to achieve durable disease control with ADT resumption as the only active agent in further line of treatment [24]. In detail, three out of four patients showed a 50% PSA decrease and in one case a radiological response was reported. The disease control duration to LHRH-A re-introduction in this small series was 4, 9, 14 and 28 months, respectively.

This original, though anecdotal, observation suggests that testosterone recovery could restore sensitivity to previous AR targeted therapies. As a matter of fact, three among the aforementioned non-randomized studies reported on the efficacy results of enzalutamide and abiraterone rechallenge upon progression to BAT and observed a PSA response ranging from 16 to 88%, and a PFS ranging from 4 to 6 months [19].

Furthermore, in the TRANSFORMER trial about 40% of patients randomized to BAT vs enzalutamide crossed over to the alternative treatment at progression, allowing an explorative comparison between the two different sequences. Interestingly, patients who received the treatment sequence of BAT followed by enzalutamide had significantly longer cumulative PFS than the opposite sequence (28.2 vs 19.6 months).

A comprehensive list of published data reporting the efficacy of ADT/NGHA rechallenge after testosterone restoration is depicted in Table 1.

**Table 1:** Results of rechallenge with hormonal agents after testosterone restoration in mCRPC patients within clinical trials

Trial	Setting	Modality of testosterone restoration	Hormonal strategies adopted after testosterone restoration	No. of patients	Outcomes
Bedussi et al. 2015 (case series)	2nd line mCRPC	Withdrawal of androgen deprivation therapy in association with Docetaxel	LHRH-analog	4	PSA50: 75% (3 patients) ORR: 25% (1 patient) DCR: 75% (3 patients)*
Schweizer et al. 2015 (pilot study)	1st Line mCRPC	Testosterone therapy according to a BAT schedule	Abiraterone, Enzalutamide, Bicalutamide, Nilutamide	10	PSA50: 70%
Teply et al. 2018 (non randomized phase II study)	2nd/3rd line mCRPC	Testosterone therapy according to a BAT schedule	Enzalutamide	21	PSA50: 52% (33–71) ORR: 0% PSA-PFS: 5.5 months (4.6-NR) crPFS: 4.7 months (2.7-NR)
Markowski et al. 2020 (non randomized phase II study)	3rd line mCRPC	Testosterone therapy according to a BAT schedule	Abiraterone, Enzalutamide	59	PSA50: 16–68% crPFS: 4–6 months PFS2: 8.1–12.8 months
Denmeade et al. TRANSFORMER 2021 (randomized phase II study)	2nd line mCRPC	Testosterone therapy according to a BAT schedule	Enzalutamide	37	PSA50: 77.8% ORR: 28.6% PSA-PFS: 10.9 months PFS2: 28.2 months vs 19.6 months**

*Duration of response: 9, 14 28+ months

**BAT-enzalutamide vs enzalutamide-BAT sequence

*PSA50* proportion of patients achieving PSA reduction ≥50%, *ORR* objective response rate, *DCR* disease control rate, *PSA-PFS* time from treatment initiation to first confirmed PSA increase, *crPFS* time from treatment initiation to clinical deterioration or radiographic progression, *PFS2* time from study initiation to progression to second line treatment, *OS* overall survival.

Ongoing studies are testing the association between BAT and chemotherapy (carboplatin, NCT03522064), immunotherapy (nivolumab, NCT03554317) and PARP-inhibitors (olaparib, NCT03516812).

The biological rationale of combo therapies is based on the acknowledgement that rapidly fluctuating testosterone levels may cause DNA breaks and genomic instability, a condition that can be exploited by treatments targeting DNA or neoantigens [25].

Preliminary results of BAT plus nivolumab/olaparib phase II trials have been recently presented, with encouraging PSA50 rate of 40–47% in similar CRPC pre-treated patient populations [26, 27]. Of note, in the olaparib combination PSA response as well as objective response were independent from DNA damage repair gene mutational status, suggesting a synergistic activity of BAT with PARP inhibition.

These data deserve to be validated within randomized phase III trials aimed at finding the optimal setting (first vs further treatment lines), schedule (intermittent vs continuous) and possible companion drug for testosterone administration in CRPC patients.

In conclusion, evidence suggests that serum testosterone has a positive prognostic role in CRPC, so ADT maintenance in CRPC patients receiving concomitant AR independent therapies is questionable. Indeed, the observation of a successful rechallenge with hormonal agents after a transient restoration in testosterone levels claims for the possibility to expand efficacy of AR-targeted therapies in CRPC setting, with relevant implications for clinical practice since these agents are currently being used in the early management of hormone-sensitive disease. In order to achieve rapid testosterone recovery in CRPC patients, ADT discontinuation alone is not sufficient and hormonal replacement is required.

Testosterone can positively influence the patients’ quality of life, which is a key clinical endpoint in late CRPC.

Whether testosterone supplementation, in association with active antineoplastic therapies for patients with CRPC, should be administered continuously to achieve stable testosterone levels within normal limits or follow the BAT schedule is a matter for future research.
